# Physical Activity, Cognitive Health and Learning in Youth: A Narrative Umbrella Review

**DOI:** 10.3390/ijerph23010011

**Published:** 2025-12-20

**Authors:** Sven Unterguggenberger

**Affiliations:** Department of Human Movement Sciences, Sport and Health, University of Graz, 8010 Graz, Austria; sven.unterguggenberger@uni-graz.at

**Keywords:** physical activity, executive functions, attention, academic performance, children and adolescents, school-based interventions

## Abstract

**Highlights:**

**Public health relevance—How does this work relate to a public health issue?**

- Physical activity shows small cognitive benefits in youth.
- Findings relate to attention, executive functions, and learning readiness.

**Public health significance—Why is this work of significance to public health?**

- Evidence is inconsistent due to major methodological variation.
- The review highlights limitations in intervention design and cognitive assessment.

**Public health implications—What are the key implications or messages for practitioners, policy makers and/or researchers in public health?**

- Schools may benefit from structured, cognitively meaningful physical activity.
- Standardized protocols are needed to guide policy and practice.

**Abstract:**

Although physical activity has been widely investigated for potential cognitive and academic benefits in children and adolescents, the evidence base remains mixed and characterized by substantial methodological variability. The present narrative umbrella review synthesizes and critically evaluates recent meta-analyses to identify patterns, strengths, and limitations in the existing literature. A comprehensive search identified systematic reviews and meta-analyses published in recent years that examined the effects of physical activity, exercise, and sports interventions on cognitive outcomes in healthy participants aged 5 to 17 years. In total, seven meta-analyses, covering 181 primary studies with approximately 42,000 participants, met the inclusion criteria. Across reviews, small positive effects of physical activity were reported for executive functions and attention, whereas findings for academic performance were inconsistent. Substantial variation in intervention duration, exercise modality, intensity, implementation context, and cognitive assessment procedures limited comparability and constrained interpretability. Overall, the synthesis indicates modest cognitive benefits of physical activity in youth, while underscoring the need for clearer operationalization of cognitive constructs, standardized intervention protocols, and transparent methodological reporting. From an applied perspective, integrating feasible, developmentally appropriate, and cognitively engaging physical activity into school and community settings may help support attention, executive functions, and broader cognitive health in young populations.

## 1. Introduction

Physical activities such as sports and non-competitive movement activities are widely recognized for their positive effects on physical and psychological health, including well-documented benefits for the cardiovascular system [1,2]. Beyond these established physiological effects, public-health research increasingly highlights the relevance of movement behaviors for cognitive development and overall wellbeing. Consequently, recent research has examined the effects of physical activity on executive functions, attention, and academic performance in children and adolescents [3].

Across existing systematic reviews and meta-analyses, several studies report small but positive effects of physical activity on executive functions and attention, particularly in inhibition and working memory [3,4]. Some analyses also indicate potential benefits for academic performance, although these findings vary notably across cognitive domains and school-related outcomes [4,5]. At the same time, multiple reviews document mixed or non-significant effects depending on intervention design, activity type, and methodological quality [6,7,8].This pattern of inconsistent outcomes aligns with broader umbrella evidence provided by Ciria et al. [9], who synthesized 24 meta-analyses of randomized controlled trials across the lifespan and reported substantial heterogeneity in cognitive domains, contributing to the overall inconclusive nature of findings in the field. The analysis included studies with aerobic and anaerobic exercise interventions lasting at least two weeks conducted with healthy participants across the lifespan and excluded studies with mixed or undefined training types. Of the 24 included reviews, only four (16%) examined children or adolescents, while approximately 70% focused on older adults. Under these inclusion criteria, Ciria et al. [9] found no consistent evidence for a causal relationship between physical activity and cognitive outcomes and suggested that effects reported in earlier reviews may have been overestimated. However, the strong emphasis on adult and older-adult samples limits the transferability of these findings to developmental contexts, where physical activity may interact differently with cognition.

While the umbrella review by Ciria et al. [9] provides a valuable synthesis of the available meta-analyses, its age distribution limits the generalizability of findings to younger populations. Furthermore, its broad scope across all age groups, intervention types, and cognitive domains leaves open important questions regarding developmental specificity. Consequently, the cognitive effects of physical activity and sports interventions in children and adolescents remain only partially understood, particularly regarding executive functions, attention, and academic performance within educational contexts.

This gap is highly relevant, as these cognitive functions are not only relevant for learning and academic performance but also represent core components of mental health and overall wellbeing in youth. Recent umbrella evidence indicates that physical activity and fitness are consistently associated with better cognitive health in children and adolescents, with cognitive functioning demonstrating one of the stronger causal indicators among youth mental-health outcomes [2]. Highlighting this broader relevance situates physical activity not only as an educational resource but also as a potential contributor to health promotion. Positioning executive functions and attention as both cognitive and mental-health determinants strengthens the rationale for investigating exercise–cognition relationships in young populations. Framing exercise–cognition relationships within this wider perspective provides a meaningful rationale for scalable implementation in school and community settings.

Despite growing evidence, findings remain inconsistent, and methodological differences across reviews complicate interpretation. Therefore, the present umbrella review aims to systematically summarize and critically evaluate meta-analyses published in recent years that examined the effects of physical activity, exercise, and sports interventions on cognitive outcomes in children and adolescents aged 5 to 17 years. Specifically, it addresses executive functions (inhibition, working memory, cognitive flexibility, and planning), attention, and academic performance, and discusses methodological sources of heterogeneity across reviews, including intervention duration, intensity, and study design characteristics. Given that all included sources are themselves systematic meta-analyses with considerable methodological variability, a narrative umbrella review approach is appropriate to integrate findings qualitatively and to capture conceptual and methodological nuances that cannot be meaningfully summarized through quantitative aggregation.

## 2. Materials and Methods

### 2.1. Study Design

This narrative umbrella review synthesized systematic reviews and meta-analyses that examined the relationship between physical activity and cognitive functions in children and adolescents. The literature search initially covered the period from 2003 to 2023 to ensure a comprehensive overview of developments over the past two decades. However, only reviews published between 2017 and 2023 met the inclusion criteria for the final synthesis, reflecting the aim to focus on recent high-quality evidence within a narrative synthesis framework. Limiting the synthesis to this period ensured that the included reviews followed comparable methodological and reporting standards. The review compares methodological characteristics, thematic focuses, and key findings across included meta-analyses.

### 2.2. Search Strategy

A comprehensive literature search was conducted in the electronic databases PubMed, Scopus, and Web of Science. In addition, ERIC (Education Resources Information Center) and Google Scholar were screened to identify further relevant publications and potential gray literature. However, only peer-reviewed systematic reviews and meta-analyses were included in the final synthesis. The search period covered January 2003 to March 2023 to capture both early foundational and more recent reviews. Only peer-reviewed publications written in English were included.

To ensure methodological consistency and to focus on the most up-to-date evidence, the detailed analysis was limited to systematic reviews published between 2017 and 2023. Earlier meta-analyses [10,11,12,13,14] addressing the same age range were included for contextual reference but excluded from the detailed synthesis due to limited temporal relevance. Publications appearing after March 2023 were not systematically screened, but were considered narratively when they complemented or contextualized the main findings.

The search strategy used combinations of keywords and Boolean operators such as: (“physical activity” OR “exercise” OR “sport” OR “fitness training”) AND (“cognition” OR “executive functions” OR “attention” OR “academic performance”) AND (“meta-analysis” OR “systematic review” OR “umbrella review”). All search terms were entered in English, with synonyms and truncations (e.g., cognit**) applied where appropriate.

### 2.3. Inclusion and Exclusion Criteria

Eligible studies were systematic reviews, meta-analyses, or umbrella reviews investigating the effects of physical activity, exercise, or sports participation on cognitive outcomes in healthy children and adolescents (5–17 years). Studies were required to report information on sample characteristics, intervention type, and cognitive outcome measures, as well as quantitative or qualitative results (Table 1).

Narrative reviews without a systematic search strategy [15,16,17,18,19], studies in which children or adolescents were analyzed only as subgroups within broader age ranges [20,21,22,23,24], and reviews focusing exclusively on pathological populations (e.g., ADHD, autism, dementia) were excluded.

Reviews published before 2017 were excluded from the detailed synthesis because methodological standards and reporting frameworks have changed considerably over time. Limiting the review to recent publications ensured better comparability and methodological consistency across the included studies.

To increase transparency in the screening process, Table 2 provides an overview of all reviews identified during the search that were excluded from the detailed synthesis, along with the primary reason for exclusion. After this screening process, seven meta-analyses met all eligibility criteria and were included in the final narrative synthesis.

**Table 1:** Included reviews and key characteristics.

Author (Year)	Study Design	Population	Included Studies (*n*)
de Greeff et al. (2018) [4]	Systematic review + meta-analysis	Children 6–12	31
Paschen et al. (2019) [6]	Systematic review	Children 5–11	10
Xue et al. (2019) [3]	Systematic review + meta-analysis	Children 6–17	19
Amatriain-Fernández et al. (2021) [25]	Systematic review + meta-analysis	Children 7–16	10
Meli et al. (2021) [5]	Systematic review + meta-analysis	Children 6–11	6
Peiris et al. (2022) [7]	Systematic review + meta-analysis	Children 7–12	10
Vasilopoulos et al. (2023) [8]	Systematic review + meta-analysis	Children 5–12	92

### 2.4. Data Extraction and Synthesis

For each included review, key information was extracted: author(s), year of publication, sample size and age range, type of intervention, cognitive domain(s) assessed, number and type of primary studies, main results, and reported effect sizes. Data were compiled in a structured Excel sheet (Microsoft Excel, Version 16.0) and qualitatively synthesized to identify overarching patterns across studies. Given the heterogeneity of cognitive constructs, intervention modalities, and analytical strategies across reviews, a purely qualitative synthesis was deemed most suitable for integrating findings.

### 2.5. Assessment of Quality and Bias

The methodological quality of the included systematic reviews and meta-analyses was assessed using the AMSTAR 2 tool [26]. AMSTAR 2 comprises 16 domains that evaluate key aspects of review quality, including protocol registration, adequacy of the search strategy, duplicate study selection and data extraction, justification of excluded studies, reporting transparency, risk-of-bias assessment, appropriateness of meta-analytic methods, and consideration of bias in the interpretation of findings. Each review was appraised across all 16 domains, and ratings were documented using the categories “Yes”, “Partial Yes”, “No”, or “Not applicable”.

Because the included reviews differed substantially in design, scope, and analytical approaches, AMSTAR 2 domain ratings were reported individually rather than aggregated into an overall score. This approach aligns with current recommendations for umbrella reviews, where methodological heterogeneity precludes meaningful summary ratings. The complete domain-level appraisal for all included reviews is presented in Table 3 and was used to contextualize methodological strengths, limitations, and comparability of the synthesized evidence.

### 2.6. Reporting Standards

Reporting of the review followed general principles of transparent research synthesis. As the present work represents a narrative umbrella review rather than a systematic review, no formal PRISMA flowchart was created.

## 3. Results

### 3.1. Overview of Included Meta-Analyses

Seven meta-analyses published between 2018 and 2023 met the inclusion criteria and collectively synthesized findings from 181 primary studies investigating the effects of physical activity and sports interventions on cognitive and academic outcomes in children and adolescents. The included reviews originated from different countries, including The Netherlands, Germany, China, Hong Kong, Malaysia, and UK, and varied considerably in their methodological design, scope, and participant characteristics [3,4,5,6,7,8,25].

Across studies, the number of included primary trials ranged from nine [5] to ninety-two [8], encompassing a total sample of approximately 42,000 children and adolescents aged between 5 and 17 years. All reviews focused on typically developing, healthy participants. Intervention formats ranged from single-session activities to multi-month longitudinal programs, and most studies were conducted within educational or school-related settings.

While all reviews examined the relationship between physical activity and executive functions, their specific aims differed. Some studies investigated broad domains such as executive functions, attention, and academic performance [4,8], whereas others focused on more specific cognitive processes, such as inhibition [25] or working memory [6]. A smaller subset explored additional factors such as health behavior and micronutrient supplementation [5,7]. The substantial heterogeneity in research questions, intervention types, and methodological designs highlights the diversity of approaches in this field and underlines the importance of a narrative synthesis.

### 3.2. Synthesized Results Across Cognitive Domains

#### 3.2.1. Executive Functions

Across the included meta-analyses, executive functions were the most frequently examined outcome domain, yet findings were heterogeneous. Small but positive effects of physical activity were reported most consistently for inhibitory control and working memory, particularly in long-term interventions (e.g., Xue et al. [3]; De Greeff et al. [4]). Acute physical activity also showed beneficial effects on specific subprocesses such as processing speed in working memory [6].

However, other components of executive functioning, including cognitive flexibility and planning, yielded mixed or predominantly non-significant results across reviews [3,4,6,8]. Meta-analytic estimates for these domains tended to be small, and several analyses reported confidence intervals overlapping with zero. Reviews focusing exclusively on inhibition showed only minimal improvements [25], suggesting that effects may be highly dependent on intervention duration, cognitive engagement, and study quality.

Overall, while executive functions show the most consistent pattern of positive associations, effect sizes are typically small, and substantial methodological variability across studies complicates interpretation.

#### 3.2.2. Attention

Attention-related outcomes demonstrated a similarly mixed pattern. Acute physical activity produced small-to-moderate improvements in attention in some analyses [4], whereas long-term interventions showed no consistent benefits across reviews [8].

Studies examining classroom-based physical activity breaks (IcPAB) reported inconsistent or null effects on attention and broader cognitive outcomes [7], despite improvements in physical activity behavior. Conversely, analyses incorporating combined interventions, such as physical activity with micronutrient supplementation, found moderate improvements in attentional capacity [5].

Taken together, attention appears responsive to acute bouts of exercise, while evidence for sustained improvements through long-term programs remains inconclusive.

#### 3.2.3. Academic Performance

The effects of physical activity on academic performance were generally limited and domain-specific. Some studies reported small positive effects on overall academic achievement [4] or substantial gains in specific subjects, such as spelling or foreign language learning [7], and mathematics [5].

However, most meta-analyses found no consistent improvements in core subjects such as mathematics and reading, and reviews covering broader age ranges or multiple cognitive domains reported predominantly null findings [8]. Academic outcomes also appeared highly sensitive to intervention type and classroom integration, with “In-classroom physical activity breaks” (IcPAB) interventions yielding benefits only in isolated cases.

Overall, academic performance shows the weakest and least consistent evidence base among the cognitive domains examined.

#### 3.2.4. Creativity and Task-Related Behavior

A smaller subset of reviews examined additional cognitive and behavioral outcomes. One review reported moderate improvements in creativity following movement-based interventions [8], although the evidence base was limited. The same review found a large but imprecise effect on task-related behavior, suggesting potential relevance for classroom engagement and learning readiness.

These findings highlight promising but under-researched domains that warrant further investigation.

### 3.3. Results by Cognitive and Intervention Characteristics

#### 3.3.1. Cognitive Variables

Across the reviewed publications, strong parallels were observed in the cognitive variables assessed (Table 4). All meta-analyses examined the effects of physical activity on executive functions, although the specific components analyzed varied depending on study design and inclusion criteria.

Inhibitory control was investigated in six reviews [3,4,6,7,8,25], while working memory was included in four [3,4,6,8], as were attention [3,4,6,8] and cognitive flexibility or planning [4,5,7,8]. Fluid intelligence was addressed in two reviews [7,8], and semantic memory was included in one [7]. Creativity and task-related behavior were analyzed exclusively in Vasilopoulos et al. [8]. Academic performance, most often represented by mathematics and reading competencies, was examined in four meta-analyses [4,5,7,8].

Overall, the included reviews focused predominantly on executive and attentional domains, while broader cognitive constructs such as creativity, fluid intelligence, or semantic memory were rarely investigated.

#### 3.3.2. Characteristics of Physical Activity Interventions

In contrast to the relative homogeneity observed in the cognitive variables, the physical activity interventions analyzed across the included meta-analyses were highly heterogeneous. Considerable variation was found in terms of exercise type, duration and intensity, and implementation context (Table 5).

In De Greeff et al. [4], neither the specific type of intervention nor the organizational conditions were described in detail. The duration of interventions was broadly categorized into short-term and long-term exercise programs, without further specification. With the exception of Paschen et al. [6], all other reviews examined the effects of long-term or longitudinal interventions. Intervention periods ranged from a single session to eight months [7], from four weeks to nine months [5], from six weeks to two years [25], and from six to fifty-four weeks [3]. Vasilopoulos et al. [8] used intervention duration as a moderator variable, classifying studies into subgroups of ≤6 weeks, 7–10 weeks, 11–24 weeks, and ≥25 weeks.

The type of physical activity was defined with varying precision across reviews. Aerobic or cardiovascular endurance activities were the most common intervention type, appearing in five of the seven reviews [3,6,7,8,25]. Six reviews included studies with coordinative exercise components or interventions incorporating at least partial coordinative demands [3,6,7,8,25]. In De Greeff et al. [4], studies combining both aerobic and coordinative exercise were excluded. The content of the included interventions was equally diverse, ranging from circuit-based endurance training, running, and cycling activities to games and team sports [6]; dance and holistic movement programs [8]; high-intensity interval training (HIIT) [25]; juggling and football [5]; and tennis or strength training [3].

Regarding the organizational setting, substantial diversity was also observed. Many interventions were implemented within regular school curricula, as part of scheduled physical education classes or integrated into other subjects as movement-oriented sessions. Peiris et al. [7] included studies that implemented in-classroom physical activity breaks (IcPAB), often through video-based exercise prompts. Meli et al. [5] also analyzed “activity breaks” embedded in classroom instruction. Xue et al. [3] included both curricular and extracurricular activities such as home-based or after-school exercise programs. Vasilopoulos et al. [8], in contrast, categorized interventions by the professional qualification of the instructor rather than by organizational setting.

In summary, the reviewed meta-analyses revealed a wide range of intervention types, durations, and implementation formats, illustrating the considerable methodological and contextual heterogeneity across studies.

## 4. Discussion

### 4.1. Executive Functions

Executive functions emerged as the most consistently examined cognitive domain across the included meta-analyses, yet the strength and reliability of the reported effects remained limited. Several reviews identified small but positive associations between physical activity and inhibitory control or working memory, particularly in long-term interventions [3,4]. Acute exercise bouts also appeared to facilitate specific subprocesses such as processing speed in working memory [6].

However, broader executive function components, namely cognitive flexibility and planning, yielded mixed or predominantly non-significant effects across reviews [3,4,6,8]. Reviews focusing exclusively on inhibition found only minimal improvements [25], suggesting that potential benefits may depend on intervention characteristics such as duration, exercise type, and cognitive engagement.

Taken together, the evidence indicates that executive functions may respond positively to physical activity, but effect sizes remain small and the confidence of conclusions is limited by methodological heterogeneity and the predominantly low to moderate quality of the included reviews according to the AMSTAR-2 criteria.

### 4.2. Attention

Attention-related outcomes showed similarly inconsistent findings. Acute physical activity produced small-to-moderate improvements in attentional performance in some analyses [4], and moderate effects emerged when physical activity was combined with micronutrient supplementation [5]. In contrast, long-term interventions and school-based activity programs revealed no consistent benefits across reviews [7,8].

Given the central role of attentional control for learning and classroom engagement, even modest improvements may be meaningful. However, the available evidence does not yet support a reliable or sustained effect of physical activity on attention.

### 4.3. Academic Performance

The effects of physical activity on academic performance were generally weak and domain-specific. While isolated studies reported positive effects on spelling, foreign language learning [7], mathematics [5], or combined indicators of academic achievement [4], most analyses found no consistent improvements in core school subjects.

A notable limitation concerns the considerable variability in the assessment instruments used to evaluate academic competencies. Only one review provided transparent information on test batteries and scoring procedures [8]. As highlighted by Peiris et al. [7], inconsistent measurement quality and a lack of standardized academic testing likely contributed to the heterogeneous findings. Overall, the evidence does not support the assumption that physical activity interventions produce reliable improvements in academic performance.

Beyond differences in measurement tools and training modalities, several additional factors may help explain the inconsistent findings for academic outcomes. Academic performance is strongly dependent on developmental stage, and teacher-related influences such as instructional quality and the ability to implement cognitively meaningful activity may affect results independently of the intervention. Moreover, physical activity may influence academic achievement indirectly through improvements in attention, classroom behavior or motivation, which are not consistently captured in the reviewed studies.

### 4.4. Creativity and Task-Related Behavior

Only one review examined creativity and task-related behavior in detail [8]. Moderate improvements in creativity and large but imprecise effects on task-related behavior suggest that these domains may hold promise for future research, particularly given their relevance for learning readiness and classroom engagement.

However, these outcomes were assessed in a very limited number of primary studies, restricting the generalizability of findings.

### 4.5. Intervention Design and Training Characteristics

Across all included meta-analyses, the most substantial source of inconsistency arose from the sport-scientific characteristics of the interventions. The primary studies encompassed fundamentally different training modalities, ranging from aerobic endurance tasks and low-intensity activity breaks to coordinative exercises, dance programs, strength-endurance circuits, and sport-specific skill activities [3,4,5,6,7,8,25]. These modalities differ in physiological load as well as in their cognitive, coordinative, and motivational demands, which limits the extent to which their effects can be meaningfully compared.

Marked discrepancies in training duration and frequency, extending from single-session interventions [7] to multi-year programs [3], further imply entirely different adaptation potentials. Such variability challenges the application of core sport-scientific principles such as specificity, overload, and progression, and likely contributes to the heterogeneity observed across cognitive outcomes.

A recurring limitation across the primary studies was the limited methodological transparency of exercise reporting. Many interventions were described using broad categories (e.g., “aerobic exercise”, “resistance training”, “coordination tasks”) without specifying intensity, dosage, structure, or instructional methods. Consequently, only a small subset of studies provided sufficient information to evaluate the underlying training rationale or allow replication. This lack of operationalization restricts the interpretability of the reported cognitive effects and represents a central deficit of the existing evidence base.

Organizational factors amplify these challenges. School-based interventions depend on teacher expertise, classroom management, and structural constraints, whereas home-based or extracurricular programs rely heavily on participant motivation and self-regulation. These contextual aspects influence whether intended training intensities or cognitive demands are actually achieved during implementation.

Taken together, these observations illustrate a key issue: the current evidence base synthesizes cognitive outcomes from interventions that differ substantially in their underlying sport science foundations and training principles.

This discrepancy between intervention design and evaluation context likely contributes to the small and inconsistent cognitive effects reported across reviews.

### 4.6. Implications

Taken together, the reviewed evidence suggests that physical activity can support executive functions and attention under certain conditions, although the magnitude and consistency of effects vary across studies. Overall, the confidence of the synthesized evidence remains limited, as the majority of included reviews were classified as low or critically low in methodological quality based on the AMSTAR-2 appraisal. Potential benefits appear closely linked to intervention quality and methodological clarity, underscoring the need for cautious interpretation when transferring findings to educational or public-health contexts. Advancing the field will require more standardized training protocols, clearer operationalization of cognitive constructs, and improved transparency in intervention reporting.

## 5. Conclusions

This narrative umbrella review synthesizes recent meta-analytic evidence on the cognitive effects of physical activity in children and adolescents. Across executive functions, attention, academic performance, and less frequently studied domains such as creativity and task-related behavior, effects were generally small and varied across studies. Differences in intervention characteristics, methodological reporting, and measurement approaches contributed substantially to these variations. While physical activity shows promise for enhancing selected cognitive processes—particularly executive functions and attentional control—the current evidence base remains evolving. More standardized protocols, consistent assessment tools, and rigorous methodological practices are needed to enable clearer conclusions and more robust recommendations for educational and public-health applications.

## Figures and Tables

**Table 2:** Excluded reviews from the detailed synthesis and corresponding reasons for exclusions.

Author(s), Year	Reason for Exclusion
Sibley & Etnier (2003) [10]	Outside analysis window
Best (2010) [11]	Non-systematic review
Haapala (2012) [12]	Non-systematic review
Chang et al. (2012) [24]	Population mismatch
Verburgh et al. (2013) [13]	Outside analysis window
Tomporowski et al. (2015) [18]	Non-systematic review
Donnelly et al. (2016) [14]	Outside analysis window
Ludyga et al. (2016) [23]	Population mismatch
Khan & Hillman (2014) [19]	Non-systematic review
Bidzan-Bluma & Lipowska (2018) [16]	Non-systematic review
Singh et al. (2018) [17]	Non-systematic review
Pontifex et al. (2019) [22]	Population mismatch
Haverkamp et al. (2020) [21]	Population mismatch
Ruhland & Lange (2021) [15]	Non-systematic review
Ishihara et al. (2021) [20]	Population mismatch

**Table 3:** AMSTAR 2 appraisal of the included systematic reviews and meta-analyses.

AMSTAR 2 Domain		de Greeff et al. [4]	Peiris et al. [7]	Paschen et al. [6]	Vasilopoulos et al. [8]	Amatriain-Fernández et al. [25]	Meli et al. [5]	Xue et al. [3]
1	PICO	Yes	Yes	Yes	Yes	Yes	Yes	Yes
2	PROT	No	Yes	No	Yes	Yes	Yes	No
3	DESIGN	Yes	Yes	Yes	Yes	Yes	Yes	Yes
4	SEARCH	No	No	No	Yes	Partial Yes	Partial Yes	Partial Yes
5	SEL.DUP	No	Yes	Yes	Yes	Yes	Yes	Yes
6	EXT.DUP	No	Yes	No	Yes	Yes	Yes	Yes
7	EXCL.JUST	Partial Yes	Yes	No	No	Partial Yes	No	No
8	CHAR	Yes	Yes	Yes	Yes	Yes	Partial Yes	Partial Yes
9	RoB.METH	Partial Yes	Yes	Yes	Yes	Yes	Partial Yes	Partial Yes
10	FUND	No	No	No	No	No	No	No
11	META.METH	Yes	Yes	Not applicable	Yes	Yes	No	No
12	RoB.META	No	Yes	Not applicable	Yes	No	No	No
13	RoB.INT	No	Yes	Yes	Yes	Yes	No	No
14	HET	Yes	Yes	Yes	Yes	Yes	No	No
15	PUB.BIAS	Yes	Yes	Not applicable	Yes	Yes	No	Yes
16	COI	Yes	Yes	No	Yes	Yes	Yes	Yes

Abbreviations: PICO = PICO clearly defined; PROT = protocol registered; DESIGN = study designs explained; SEARCH = adequate search strategy; SEL.DUP = study selection in duplicate; EXT.DUP = data extraction in duplicate; EXCL.JUST = justification of excluded studies; CHAR = study characteristics reported; RoB.METH = risk-of-bias assessment methods; FUND = funding reported; META.METH = appropriateness of meta-analytic methods; RoB.META = risk-of-bias in meta-analysis; RoB.INT = risk-of-bias in interpretation; HET = heterogeneity assessment; PUB.BIAS = publication bias assessment; COI = conflict of interest reported; NA = not applicable.

**Table 4:** Cognitive variables and outcome domains examined in the included meta-analyses.

Cognitive Variable	de Greeff et al. (2018) [4]	Paschen et al. (2019) [6]	Xue et al. (2019) [3]	Amatriain-Fernández et al. (2021) [25]	Meli et al. (2021) [5]	Peiris et al. (2022) [7]	Vasilopoulos et al. (2023) [8]
Inhibition	✓	✓	✓	✓	–	✓	✓
Working memory	✓	✓	✓	–	–	–	✓
Cognitive flexibility/planning	✓	✓	✓	–	–	–	✓
Attention	✓	–	–	–	✓	✓	✓
Fluid intelligence	–	–	–	–	–	✓	✓
Semantic memory	–	–	–	–	–	✓	–
Creativity	–	–	–	–	–	–	✓
Task-related behavior	–	–	–	–	–	–	✓
Academic performance (math /reading)	✓	–	–	–	✓	✓	✓

**Table 5:** Characteristics of physical activity interventions across included meta-analyses.

Study	Duration Range	Study Design	Type of Exercise	Coordinative Elements	Organizational Context/Setting
De Greeff et al. (2018) [4]	Short-term vs. long-term (unspecified)	RCTs	Not specified (aerobic or cognitive separated)	No (exclusive classification)	Not specified
Paschen et al. (2019) [6]	Acute	RCTs (within/between-subject)	Cardiovascular (low cognitive demand); coordinative (high cognitive demand)	Yes	Laboratory/controlled school settings
Xue et al. (2019) [3]	6–54 weeks	RCTs	Aerobic and strength exercises	Yes	Curricular and extracurricular (including home-based)
Amatriain-Fernández et al. (2021) [25]	≥6 weeks–2 years	RCTs	Aerobic and HIIT training	Yes	School-based
Meli et al. (2021) [5]	4 weeks–9 months	RCTs	Aerobic and coordinative activities (e.g., football, juggling)	Yes	Classroom “activity breaks”
Peiris et al. (2022) [7]	1 day–8 months	RCTs	Classroom-based physical activity breaks (IcPAB)	Partial	In-class, video-guided sessions
Vasilopoulos et al. (2023) [8]	≤6 to ≥25 weeks (duration moderator)	RCTs	Aerobic, dance, holistic movement	Yes	Grouped by instructor qualification

Abbreviations: RCT = Randomized Controlled Trial; HIIT = High-Intensity Interval Training; IcPAB = In-classroom Physical Activity Break(s).

## Data Availability

No new data were created or analyzed in this study. Data sharing is not applicable to this article.

## Abbreviations

The following abbreviations are used in this manuscript:

CI	Confidence interval
EF	Executive functions
HIIT	High-intensity interval training
IcPAB	In-classroom physical activity breaks
I^2^	Heterogeneity statistic (I-squared)
MVPA	Moderate-to-vigorous physical activity
PA	Physical activity
RCT	Randomized controlled trial
SD	Standard deviation Directory of open access journals
